# MIF inhibition attenuates proliferative vitreoretinopathy pathogenesis and protects the eye in preclinical model

**DOI:** 10.1016/j.biopha.2025.118943

**Published:** 2026-01-15

**Authors:** Sumaya Hamadmad, Tyler Heisler-Taylor, Diana Summitt, Rahaf Shalash, Ali Zatari, Dena Martini, Misha Sohail, Julie Racine, Zhiliang Xie, Kasey Hill, Mitch Phelps, Colleen M. Cebulla

**Affiliations:** a Department of Ophthalmology and Visual Sciences, Havener Eye Institute, The Ohio State University, Wexner Medical Center, Columbus, OH, USA; b Nationwide Children’s Hospital, Columbus, OH, USA; c Department of Pharmacy - Pharmaceutics and Pharmacology, The Ohio State University, Wexner Medical Center, Columbus, OH, USA

**Keywords:** Retina, Proliferative vitreoretinopathy, PVR, Retinal detachment, MIF, Ibudilast, Gliosis, AV-1013, MIF inhibition, Rabbit model, Retinopathy, Inflammation, Eye disease, Translational medicine, Animal models for eye disease

## Abstract

Proliferative vitreoretinopathy (PVR) is the leading cause of surgical failure after rhegmatogenous retinal detachment, yet no pharmacologic treatment exists. Previously, we showed that inhibiting macrophage migration inhibitory factor (MIF)—a pleiotropic cytokine implicated in inflammation and fibrosis- protects the retinal neuron structure and function in a mouse model of retinal detachment and chick model of excitotoxic damage. In the present study, we demonstrate that inhibition of MIF provides protection from PVR both *in vitro* and *in vivo*. Vitreous samples of patients with PVR revealed markedly elevated MIF levels compared with controls. In TGFβ-cultured retinal pigment epithelial cells- an established model for *in vitro* PVR- MIF inhibition reduced proliferation and migration, suppressed epithelial–mesenchymal transition, and inhibited collagen contraction, key steps in PVR progression. *In vivo*, the clinically relevant MIF inhibitor ibudilast attenuated retinal gliosis in the retina and demonstrated a favorable safety profile in both chicks and rabbits. Most importantly, in a rabbit model of PVR, intravitreal ibudilast significantly slowed disease progression, protecting eyes from advancing to higher disease grades. These findings establish MIF as a likely target for PVR treatment and position ibudilast as a promising candidate with high translational potential.

## 1. Introduction

Proliferative vitreoretinopathy (PVR) is a pathologic wound healing response that can follow retinal detachment (RD). It occurs in 5–10 % of cases and is the leading cause for failure of the surgery [1,2]. PVR is associated with poor visual outcomes and currently there are limited treatment options for PVR other than additional surgical intervention. Prevention of PVR development during the initial RD repair could lead to better visual outcomes and improve the overall success of the surgery.

PVR pathogenesis is a complex and multifactorial process that is not completely understood [3]. Inflammation, epithelial mesenchymal transition (EMT), cell proliferation, extracellular matrix disposition, and genetic factors are all thought to play a role [4]. Traditionally, the retinal pigment epithelial (RPE) cells that disperse into the vitreous cavity following the retinal break are thought to be major contributors to PVR. Exposure of these cells to the growth factors and cytokines in the vitreous leads to their proliferation, mesenchymal transition, further migration, and formation of contractile elements that cause retinal stiffening which is the hallmark of PVR [5]. Müller glia (MG), supporting cells that span the thickness of the retina, have also emerged lately as contributors to the pathophysiology of PVR [6] through secretion of cytokines and growth factors, extension of their processes [7], and upregulation of intermediate filaments (e.g., glial fibrillary acidic protein, GFAP) that leads to stiffening and re-detachment of the retina [8].

Multiple cytokines and growth factors have been shown to be upregulated in the vitreous to catalyze the above processes. We and others have identified the pro-inflammatory cytokine macrophage migration inhibitory factor (MIF) as a contributor to the pathophysiology in several retinal disease models including retinal detachment, epiretinal membrane, and excitotoxic retinal damage [9,10]. MIF has been shown to be elevated in cases of proliferative vitreoretinopathy (PVR) [11,12]. MIF plays a key role in immune system regulation, is known to upregulate pro-inflammatory cytokines [10,13,14], and is involved in multiple fibrotic conditions [15,16]. In the retina, MIF is highly expressed in the MG and can influence the release of other pro-inflammatory cytokines in a paracrine and autocrine fashion [17]. We have recently characterized many of those networks in the retina and showed that MIF inhibition can influence them during excitotoxic damage [17]. Moreover, our lab identified a human genetic association of MIF promoter polymorphisms with epiretinal membrane (ERM) formation [18]. Considering that ERM formation shares many underlying mechanisms with PVR, a role for MIF in PVR merits further investigation.

MIF inhibition has been investigated as a strategy to treat many pathologic conditions with an inflammatory component, including autoimmune disease [19], cancer [20], and neuropathy [21,22]. Both small molecule inhibitors and blocking antibodies have been utilized. We have shown that pharmacologic and genetic MIF inhibition is neuroprotective in a murine retinal detachment model [9]. We have also recently demonstrated that the clinically relevant drug, ibudilast, which acts as MIF and phosphodiesterase (PDE) inhibitor, has neuroprotective capabilities in a retinal excitotoxic model *in vivo* [17]. In this study we investigate the role of several clinically-relevant pharmacological MIF inhibitors in *in vitro* and *in vivo* PVR models that could potentially be translated to humans.

## 2. Materials and methods

### 2.1. Patients

The group of patients used in this study is part of a larger retina study group (The OVER-PVR Study) [18,23]. The study was approved by the IRB at The Ohio State University (2011H0399). The research followed the tenets of the Declaration of Helsinki. The study subjects were recruited from multiple referral-based subspecialty practices in the US as part of the OSU Vitreoretinal ERM–PVR (OVER-PVR) Study Group at The Ohio State University Wexner Medical Center in Columbus, Ohio; the Cincinnati Eye Institute in Cincinnati, Ohio; and Mary Lanning Health Care in Hastings, Nebraska. Once informed consent was obtained, a brief medical history was collected from each patient. Vitreous humor samples were collected during surgery. Epiretinal membrane (ERM) and macular hole (MH) patients were used as controls. The Retina Society Terminology Committee Classification [24] was used for PVR grading [24]. All patient and lab data were entered into REDCap Software for record collection and quality control. The vitreous of 37 PVR and 61 control (39 ERM and 22 macular hole) pseudophakic patients was analyzed by ELISA for MIF levels.

### 2.2. Animals

This research adheres to the principles of the National Institutes of Health and the ARVO Statement for the Use of Animals in Ophthalmic and Vision Research. It was conducted under a protocol approved by The Ohio State University Institutional Animal Care and Use Committee. All of the procedures were performed under anesthesia. Newly hatched wild type leghorn chicks (Gallus domesticus) were obtained from Meyer Hatchery (Polk, Ohio). New Zealand White rabbits age 6–8 weeks and weighing ~1.5–2.5 kg were acquired from Charles River Laboratories and housed in OSU animal facility under a 12-hour light:dark cycle.

### 2.3. MIF level in the vitreous

MIF level in the patients’ vitreous was measured using Elisa kit (Invitrogen, Catalog # EHMIF) according to the manufacturer’s protocol. Total protein level in the vitreous was analyzed with BCA method using BCA Protein Assay Kit (Pierce, Catalog # A55865). Normalized MIF was calculated by dividing MIF level (pg/mL) by total protein in the vitreous (ug/mL).

### 2.4. In vitro PVR model

The human RPE cell line ARPE-19 was acquired from the American Type Culture Collection (ATCC, Manassas, VA, USA). Validation of the cell line was done to confirm matching published STR profile. Cells were cultured in DMEM/F12 medium with 10 % fetal bovine serum (FBS, Gibco, Grand Island, NY, USA) 100 U/mL penicillin, 100 μg/mL streptomycin, and 0.25 μg/mL amphotericin B (Anti-Anti, Gibco, Grand Island, NY, USA). Cells were cultured in a humidified environment at 37 °C with 5 % CO2. In experiments where cells were starved 1 % FBS was used. For PVR model cells were starved for 24 hrs and then treated with 10 ng/mL TGFβ for the indicated length of time.

### 2.5. Proliferation and toxicity assays

Cell proliferation was measured using Vybrant MTT cell proliferation Assay Kit (Molecular Probes Cat No. V-13154). ARPE-19 cells were seeded at 1 × 10^4^ /cm^2^ in 96-well plate in DMEM/F12/10 % FBS for 24 h and treated with different concentrations of indicated drugs for 48 h. The substrate MTT (Roche Diagnostics Corporation, Indianapolis, IN) was added to each well for 4 h according to the manufacturer’s protocol. Then 100 μl of the lysis buffer was added to each well containing MTT. After incubation at 37°C overnight, the color development was measured by absorbance at 550 nm, with a reference absorbance at 690 nm. Each sample was assayed at least in triplicate. TUNEL assay was performed to detect apoptosis and cell death with the In Situ Cell Death Kit (TMR red, Roche Applied Science, #12156792910), as per the manufacturer’s instructions. ARPE-19 cells were cultured in 8-well chamber slides, serum starved, and treated with TGFβ. Different concentrations of the MIF inhibitors were applied for 24 h after serum starvation to assess toxicity. Hydrogen peroxide was used at 10 mg/mL concentration for 24 h to induce apoptosis as a positive control for cell death.

### 2.6. Migration assay

Cell migration assays were performed using the CytoSelect 24 well Cell migration Assay (8um, colorimetric format) kit (Cell Biolabs, Cat No. CBA-100). In summary RPE cells were serum starved overnight, then were trypsinized and suspended in serum free medium at 1.0 × 10^6^ density. TGFβ was added to the lower well of the migration plate and 300 μl of the cell suspension was added to the upper chamber (inside of the insert). When used, drugs were added to both compartments (upper and lower). After 24 hrs of incubation cells were completely removed from the upper chamber with a cotton swab. Cells on the other side of the insert were stained, extracted and transferred to a 96-well plate. The optic density at 560 nm was measured using a plate reader to quantify migratory cells.

### 2.7. Epithelial mesenchymal transition assays-immunohistochemistry

To test EMT, 8-well slides (ThermoFisher, Cat No. 12-565-18) were used to plate ARPE-19 cell at a density of 0.5–1.0 × 10^5^ cell per well. Cells were allowed to grow for one week and then serum starved for another week before the epithelial phenotype (ZO-1 expression) was established. After starvation, 10 ng/mL TGFβ was added with or without the indicated drug treatment for 24 hrs. Slides were then washed twice with PBS, fixed with 4 % paraformaldehyde (PFA) for 15 min then washed again with PBS. Before immunostaining, the cells were permeabilized in 0.25 % Triton X-100 for 10 min, rinsed in PBS, and then blocked with 1 % bovine serum albumin (BSA) for 30 min at room temperature before incubation with primary antibodies overnight at 4°C. Smooth muscle actin (SMA, Invitrogen, Cat No. MA5-11547) (1:100), vimentin (Invitrogen, Cat No. MA1-10459) (1:500), and ZO-1 (Life Technologies, Cat No. 40-2300) (1:500) antibodies were used at the indicated dilutions. The slides were next incubated with the secondary antibodies (for 1 h at room temperature. The nuclei were counterstained with DAPI (Invitrogen, Cat No. D1306). The staining was visualized and captured by Nikon confocal microscopy.

### 2.8. Gel contraction assay

Gel contraction experiments were performed using CytoSelect 24-well Cell Contraction Assay Kit (Floating Matrix Model, Cell Biolabs, Cat No. CBA-5020). Briefly RPE cells were serum starved overnight, then trypsinized and suspended in serum free medium at 3.0 × 10^6^ density. Cell suspension was mixed with collagen solution and added to the wells in the presence or absence of drugs according to the kit instructions. Collagen gel was imaged every 8 h over 2 days period and the gel size measured using Image Pro software.

### 2.9. Chick intravitreal injection

Intravitreal injection of drugs, including NMDA, ibudilast, and AV013, was delivered in 20ul volume and performed as described previously [17] using Hamilton or insulin needles and syringes.

### 2.10. Enucleation, fixation, sectioning, and immunohistochemistry

Chicks were euthanized and eyes enucleated as described previously [17]. Eyes were fixed using 4 % paraformaldehyde, embedded in OCT medium (Tissue-Tek), and freeze-mounted onto sectioning blocks. 12 μm transverse sections of the retina were cut and mounted onto slides. Sections were air-dried and stored at − 20 °C until use. For Immunohistochemistry, sections were permeabilized with 0.2 % Triton X-100 and incubated with GFAP (Invitrogen, Cat No. 53–9892) primary antibody solution overnight at 4 °C. The slides were then incubated for at least 1 h at room temperature in a humidified chamber with Alexa-fluor 488 conjugated secondary antibodies (Invitrogen, Cat No. A11055). Cell nuclei were counterstained with DAPI.

### 2.11. Ibudilast pharmacokinetic studies (Chick and Rabbit)

For the chick pharmacokinetic (PK) study, 20ul of 1.0 mg/mL ibudilast was injected at time 0 in undamaged chicks (n = 4/group). Eyes were harvested at the following timepoints: 5 min, 10 min, 15 min, 30 min, 60 min, 120 min, 180 min, 480 min, and 1440 min. Retina and vitreous were collected from each subject. Retina and vitreous from the fellow eye as well as blood were additionally collected. All tests performed on the fellow eyes and plasma were either not found or below the lower limit of quantification (BLOQ). The injected eye vitreous and retina were averaged and plotted as log ng/mL against time (min).

For the rabbit experiment, total of 12 rabbits were used to perform the PK study. Rabbits received an intraocular injection of 50 μl of 1 mg/mL ibudilast and were euthanized at the following timepoints to measure drug concentrations in the vitreous, retina, and plasma: 5, 10, 30, 60, 120, 240, and 480 min post-injection.

Ibudilast concentrations in chicken and rabbit plasma, retina, and vitreous were quantified using a liquid chromatography-tandem mass spectrometry (LC-MS/MS) method based on Sanftner, et al. [25] with modifications. The retina tissue was weighed then homogenized after the addition of 1X PBS pH 7.4 using a hand-held homogenizer (PRO Scientific Inc., Oxford, CT). Briefly, the plasma, vitreous, and retina homogenate samples were prepared with protein precipitation using cold acetonitrile after the initial addition of 500 ng/mL ibudilast-d7. The plasma and retina supernatants were dried under a stream of nitrogen then reconstituted with water: acetonitrile with 0.2 % formic acid (90:10 v/v). The vitreous supernatant was diluted with water containing 0.2 % formic acid before analysis. For all matrices a 5 μL injection volume was used. The chromatographic separation was performed on an Agilent Zorbax Extend-C18 3.5 μm, 2.1 × 50 mm column with a Thermo Aquasil C18 3 μm Javelin guard at 40 °C. using gradient elution with water and acetonitrile each containing 0.2 % formic acid. The total run time was 3.75 min. The LC-MS/MS analysis was conducted on a Thermo Vanquish UHPLC with a TSQ Quantiva mass spectrometer equipped with a heated electrospray ionization source. Nitrogen was used for the sheath and auxiliary gases and argon was used as the collision gas. Ibudilast and ibudilast-d7 were measured by selected reaction monitoring (SRM) in positive ion mode at *m/z* of 231.162 → 161.155 and 238.2 → 162.155, respectively. Xcalibur software (version 4.4.16.14) was used for instrument control and data processing. The linear range is 1–1000 ng/mL for ibudilast in chicken and rabbit plasma and vitreous and is 25–25,000 ng/g for retina in both species.

### 2.12. In vivo MIF inhibitor rabbit toxicity studies: electroretinography

Rabbit electroretinography (ERG) was performed prior to and one day after injection of ibudilast using the UTAS BigShot version 9.4.0 and the Celeris ERG with rabbit adaptor module. Rabbits that had been dark-adapted the same length of time (30 min) were anaesthetized under safe-light conditions. Eyes were dilated using both 1 % tropicamide and 2.5 % phenylephrine hydrochloride. To ensure constant body temperature during testing, animals were put on a warming source. Silver wire loop electrodes were placed on both corneas with Genteal gel to maintain corneal hydration. A gold minidisc reference electrode was placed on the tongue, and a ground needle electrode was placed subcutaneously in/near the tail. The animal’s head was positioned under the center of the Ganzfeld dome. ERG was performed for both eyes at the same time. Single flash stimuli (4 ms duration) were presented at 11 increasing intensities ranging from 0.001 to 25 cd s/m2. Five ERG traces were obtained and averaged for each luminance step. The minimum negativity occurring between 10 and 40 ms post-stimulus is defined as the a-wave. The maximum positivity occurring between 40 and 80 ms post-stimulus is defined as the b-wave.

### 2.13. Rabbit in vivo PVR model and MIF inhibition study

This PVR model was modified from Pennock et al. [26]. Rabbit conjunctival fibroblasts were prepared by harvesting the subconjunctival tissue of several rabbits. The tissues were shredded and cultured in 35-mm petri dishes containing Dulbecco’s modified Eagle’s medium (DMEM) with 10 % fetal bovine serum, gentamicin (50 g/mL), and amphotericin B as nutrient media. Confluent cultures were trypsinized, centrifuged, and resuspended in culture flasks. Platelet rich plasma (PRP) was prepared from rabbits. Blood was collected into EDTA vacuum tubes from the ear veins of rabbit. The samples were centrifuged at 180 *g* for 5 min to separate the PRP (supernatant) from the erythrocytes and leukocytes. The PRP was transferred into a clean tube, centrifuged at 200 *g* for 10 min and then preserved on ice for approximately 10 min and stored in −80 until intravitreal injection.

PVR was induced in the left eye of twenty albino rabbits following a previously described protocol [26]. Briefly rabbits were sedated with acetylpromazine (0.5–10 mg/kg) and anesthetized with isoflurane gas. Rabbits were injected with 0.05 mL of C3F8 gas using a 28-gauge insulin needle into the vitreous cavity 3 mm posterior to the limbus. One week later, each experimental eye was injected with 1 × 10^6^ primary rabbit conjunctival fibroblasts suspended in 0.1 mL rabbit PRP. Five minutes after cell injection 0.05 mL of 3 mg/mL ibudilast or saline was injected in the PVR eye. The experimental MIF inhibitor drug or vehicle injection was repeated 3 days after initial injection. Clinical examinations were performed. The animals underwent eye examinations before injection and at 1, 3, 7, 14, 21, and 28 days in a masked fashion through indirect ophthalmoscopy and slit lamp exam to evaluate for retinal hemorrhages/whitening, and infection or inflammatory response. PVR was graded according to the five-stage scale of Fastenberg described as follows: stage 0, no disease; 1, epiretinal-membrane formation; 2, vitreoretinal traction without retinal detachment; 3, localized retinal detachment of one to two quadrants; 4, extensive retinal detachment of two to four quadrants, without complete detachment; and 5, complete retinal detachment. Intraocular pressure (IOP) was checked in both eyes using the iCare Tonovet Plus under anesthesia. SD-OCT/fundus imaging was performed on a subset of rabbits using Envisu R2200 SDOIS, a rabbit bore lens, and the InVivoVue software. On day 28, animals were euthanized, and eyes were enucleated.

### 2.14. Retinal imaging

Fundus photographs and spectral domain optical coherence tomography (SD-OCT) images were taken in anesthetized and dilated animals with a Bioptigen Envisu R2200 SDOIS imaging system, a rabbit bore lens, and the InVivoVue software. Corneas were lubricated with Systane or balanced salt solution drops.

### 2.15. Statistics

Data were analyzed in a masked fashion and calculated in Microsoft Excel, JMP, and GraphPad PRISM. The *t*-test was performed to evaluate differences between experimental groups. Error bars are listed with standard error of the mean (SEM) unless stated otherwise and an α = 0.05. Experiments were run through the 4 normality tests in GraphPad Prism (D’Agostino-Pearson omnibus, Anderson-Darling, Shapiro-Wilk, and Kolmogorov-Smimov) to confirm normality of the data. When the normality tests failed, non-parametric statistics were used instead.

## 3. Results

### 3.1. MIF levels increase in vitreous samples of PVR patients

**Fig. 1.**
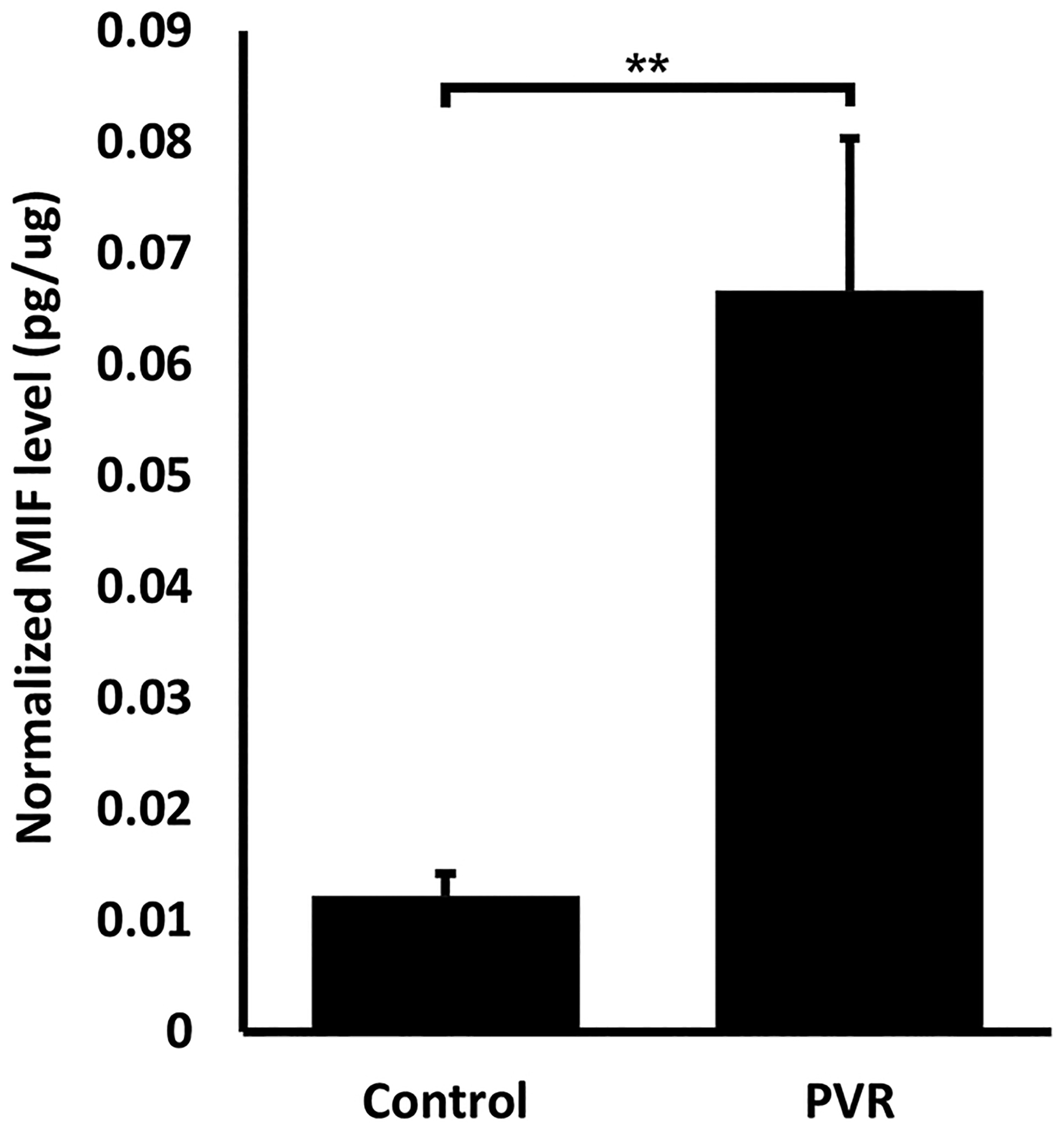
MIF level in the vitreous of PVR patients. MIF level in the vitreous of 37 PVR patients (grade B-D) and 61 controls was measured using Elisa, and total protein level in the vitreous was analyzed with BCA method. Normalized MIF was calculated by dividing MIF level (pg/mL) by total protein in the vitreous (ug/mL), *p* = 0.0014).

MIF has been implicated in the pathogenesis of different diseases including those with a fibrotic component [18,27,28]. To assess the role of MIF in PVR, we measured MIF levels in the vitreous of patients with different stages of PVR (grades B-D) and compared it to levels in the vitreous of non PVR control patients, namely macular hole and ERM. Undiluted vitreous samples were collected; ELISA was used to measure the concentration of MIF in all groups. BCA was used to measure total protein in the vitreous and MIF level was normalized to total protein level. Our results are shown in Fig. 1. Interestingly there was a statistically significant 5-fold increase in normalized MIF levels in the vitreous of patients with PVR compared to control (0.0122 vs 0.0666 pg MIF/ug total protein, *p-value* =0.0014).

### 3.2. MIF inhibition blocks RPE proliferation with no toxicity

**Fig. 2.**
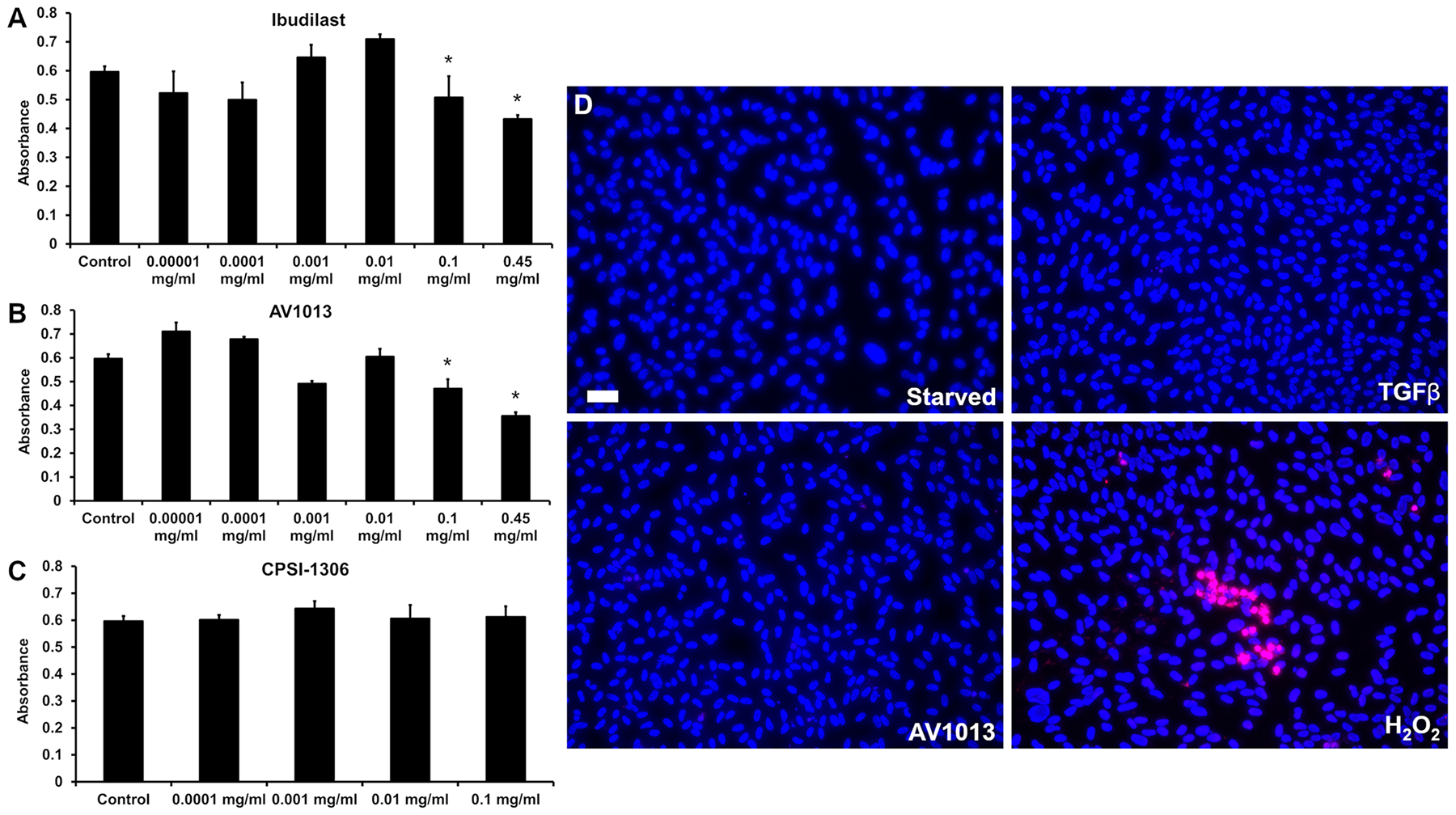
MIF inhibitors block ARPE-19 proliferation differentially without inducing toxicity. A-C. Cell proliferation was assessed in the *in vitro* PVR model using the MTT assay. ARPE-19 cells were treated with TGFb in the presence of the indicated concentrations of ibudilast (A), AV-1013 (B), and CPSI-1306 (C) for 48 hrs. MTT substrate was added according to manufacturer’s protocol and absorbance was measured after cell lysis. Asterisks indicate *p* value less than 0.05 compared to control. D. cells were treated with TGFb, AV1013 at the highest concentration (0.45 mg/mL), or H2O2, a cell toxin. Cells were fixed and stained with Dapi (blue) and TUNEL (red) to show cell death. Calibration bar = 50 μm. Graphs represent at least 3 independent experiments each done in triplicate.

Our findings that MIF levels go up in PVR patients and our previous findings that MIF inhibition can be protective in other retinal disease models [9,17] prompted us to investigate a role for MIF in the pathogenesis of PVR. To do so we used an *in vitro* model for PVR to test the effect of different MIF inhibitors on the multiple steps involved in PVR progression: namely, cell proliferation, EMT, cell migration, and matrix contraction. We selected 3 different MIF inhibitors: (i) ibudilast, a combined allosteric MIF inhibitor and phosphodiesterase-3,-4,-10, and −11 inhibitor which is used clinically in Japan and is in clinical trials in the US [29], (ii) AV1013; an analog of ibudilast that lacks the PDE activity, and (iii) CPSI-1306, a competitive isooxazoline that is derived from the prototypical MIF antagonist ISO-1 but has improved pharmacologic properties [30,31]. *In vitro* PVR was established by treating serum starved ARPE-19 cells with TGFβ, as described earlier [32]. Cell proliferation was assessed in the *in vitro* PVR model using the MTT assay (Fig. 2 A). ARPE-19 cells were treated with TGFβ in the presence of varying concentrations of ibudilast, AV-1013, and CPSI-1306. While CPSI-1306 did not have any effect on proliferation (Fig. 2 C), both ibudilast and AV1013 reduced cell proliferation in a dose-dependent manner at the 0.1–0.45 mg/mL range (Fig. 2 A, B). The ibudilast analog AV1013 had a more robust reduction of proliferation (40 % vs 27 % with ibudilast) at the highest concentration (0.45 mg/mL).

To assess potential toxicity of the drug and ensure the MTT results were not due to cell death from drug treatment, we performed TUNEL assay to quantitate dying cells after treatment. Hydrogen peroxide (H_2_O_2_), used as a positive control, induced cell death and TUNEL reactivity (Fig. 2D). In contrast, AV1013 at the dose of 0.45 mg/mL which achieved the highest level of inhibition of cell proliferation in the MTT assay, did not induce cell death. This suggests that the inhibition of cell proliferation observed in the MTT assay is not due to toxicity of the drugs.

### 3.3. MIF inhibition blocks RPE cell migration

**Fig. 3.**
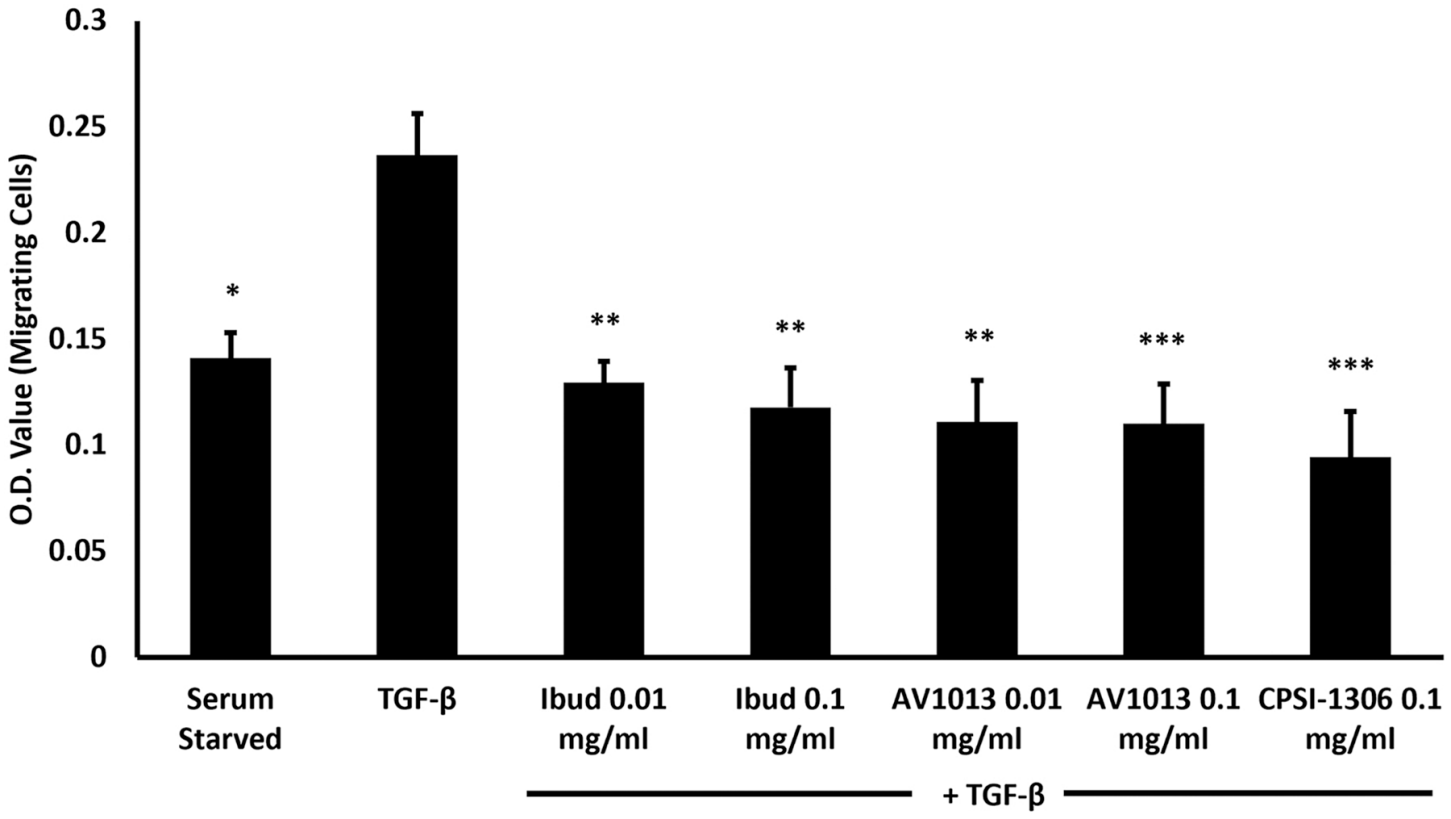
MIF inhibition effect on ARPE-19 migration. The transwell assay was used to measure TGFb-stimulated ARPE-19 cell migration. ARPE-19 cells were suspended in serum free medium at 1.0 × 106 density and added to the upper chamber and TGFb was added to the lower chamber of the migration plate (except in the starved condition, no TGFb was added). When used, drugs were added to both compartments (upper and lower). After 24 hrs of incubation cells were completely removed from the upper chamber and cells on the other side of the insert were stained and extracted. The optic density at 560 nm was measured using a plate reader to quantify migratory cells. Graphs represent 3 independent experiments each done in triplicate.

Under physiological conditions, the RPE monolayer is maintained by cell-cell contact. However, during retinal detachment and exposure of the RPE to the various growth factors and cytokines in the vitreous, the gap junction complexes are disrupted and the RPE cells migrate, contributing to PVR formation [33,34]. To investigate the impact of MIF inhibition on RPE migration, we measured TGFβ-stimulated ARPE-19 cell migration using the transwell assay in the *in vitro* PVR model. As shown in Fig. 3, TGFβ was found to enhance the migration of RPE cells; however, migration was significantly reduced by treatment with ibudilast and AV1013 at both concentrations tested (0.01 and 0.1 mg/mL). CPSI inhibited migration only at the higher concentration (0.1 mg/mL).

### 3.4. MIF inhibition impedes epithelial mesenchymal transition (EMT)

**Fig. 4.**
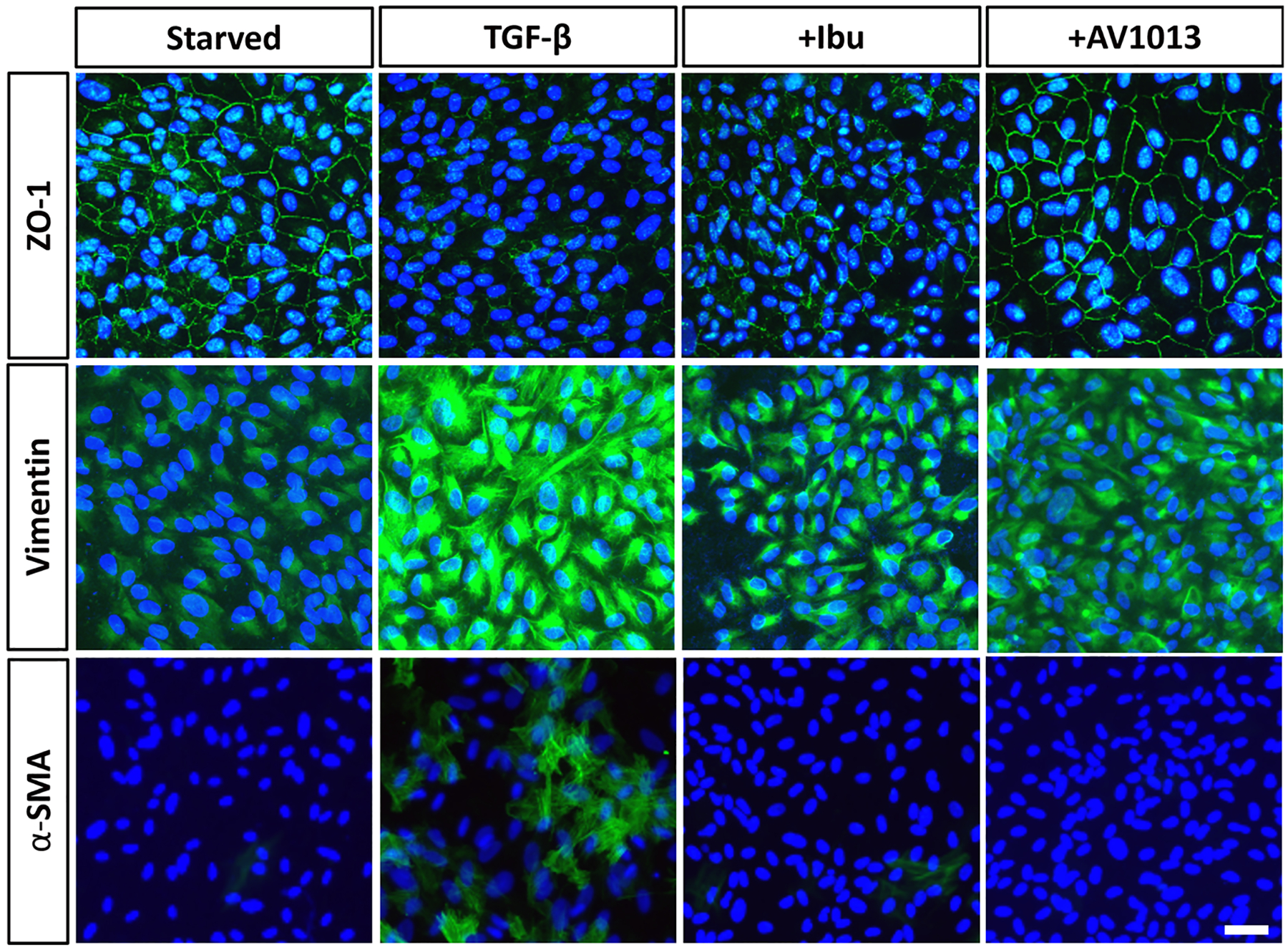
EMT markers expression in ARPE-19 cells upon MIF inhibition. Cells were seeded for 48 hrs and starved for 5–12 day afterwards. TGFb was then added with or without the indicated drug treatment for 48 hrs. Cells were fixed, permeabilized, and stained with the indicated primary antibodies (green). SMA: Smooth muscle actin and vimentin are mesenchymal phenotype markers while ZO-1 is epithelial phenotype marker. The nuclei were counterstained with DAPI (blue). The staining was visualized and captured by confocal microscopy. Images represent findings from at least 3 independent experiments. Calibration bar = 50 μm.

EMT is one of the hallmarks of PVR [34]. In this process the RPE cells lose their epithelial phenotype, which is characterized by polarity, adhesion, and gap junctions and acquire a mesenchymal, myofibroblast phenotype where they can proliferate, migrate, and contribute to fibrotic membrane formation. Cell markers for the epithelial phenotype include Zonula Occludens-1 (ZO-1) while the mesenchymal cells can be identified by expression of vimentin and α-smooth muscle actin (SMA) [35]. To elucidate the effect of MIF inhibition on EMT, markers were measured using immunofluorescence microscopy in the *in vitro* PVR model. Treatment with TGFβ induced high levels of SMA and vimentin and decreased ZO-1 levels, consistent with development of EMT (Fig. 4). Importantly, treatment with MIF inhibitors AV1013 and ibudilast reversed the TGFβ-mediated EMT effects, with increased ZO-1 and decreased SMA and vimentin expression, compared to controls. This is consistent with our finding that MIF inhibitors block all steps involved in the pathogenesis of PVR formation *in vitro*.

### 3.5. Collagen gel contraction

**Fig. 5.**
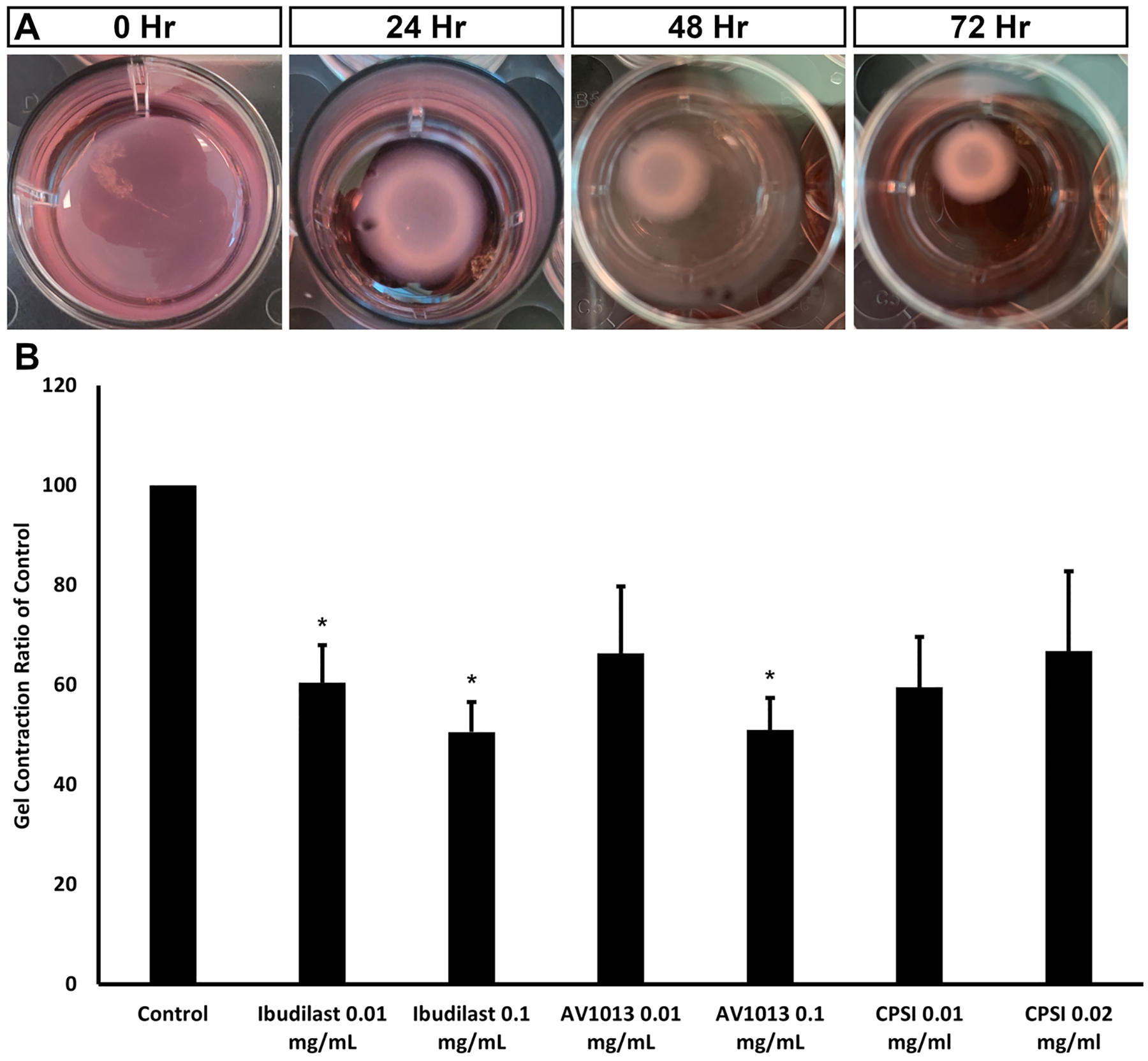
Effect of MIF inhibition on ARPE-19 gel contraction. A. ARPE cells were serum starved then suspended in TGFb medium and mixed with collagen solution in the presences or absence of the indicted MIF inhibitors. Gel contraction was assessed over 48 hr period. The images are representative of 0.1 mg/mL concentration for both drugs. B. the graph shows contraction ratio relative to TGFb control at 48 hrs which is set at 100. The gel size was measured using Image Pro software. Asterisks indicate *p* value < 0.05 compared to control (n = 11). Ibudilast 0.01 mg/mL (n = 9) p-value= 0.0003, Ibudilast 0.1 mg/mL (n = 7) *p-value* = 4.98 × 10^−08^, AV1013 0.01 mg/mL (n = 6), AV1013 0.1 mg/mL (n = 6) *p-value* = 2.08 × 10^−08^.

PVR is characterized by the formation of contractile membranes in the inner and outer surfaces of the retina, leading to re-detachment after surgical repair [36]. These membranes contain both cellular and extracellular matrix components. We sought to study cell contractability and the effect of MIF inhibitor drug on contraction in the *in vitro* PVR model using a collagen-I gel contraction assay. As Fig. 5A upper row shows, in the presence of TGFβ, ARPE-19 cells induce a time dependent gel contraction that measures 60 % at 24 hrs and 80 % at 48 hrs compared to time 0. MIF inhibition effectively reduced gel contraction (Fig. 5B), with ibudilast 0.1 mg/mL reducing contraction by 49 % (*p-value* =4.98 × 10^−08^) and AV1013 by 47 % (*p-value* =2.08 × 10^−08^) at 48 hrs. CPSI was the least effective of the 3 inhibitors, unable to show statistically significant inhibition from controls (Fig. 5C). These results implied that MIF inhibition could block the TGFβ-induced matrix contraction ability of RPE cells. Due to the lower efficacy of CPSI on *in vitro* proliferation and migration PVR studies, we decided to focus on ibudilast and AV1013 in the upcoming experiments.

### 3.6. Gliosis in chick retina

**Fig. 6.**
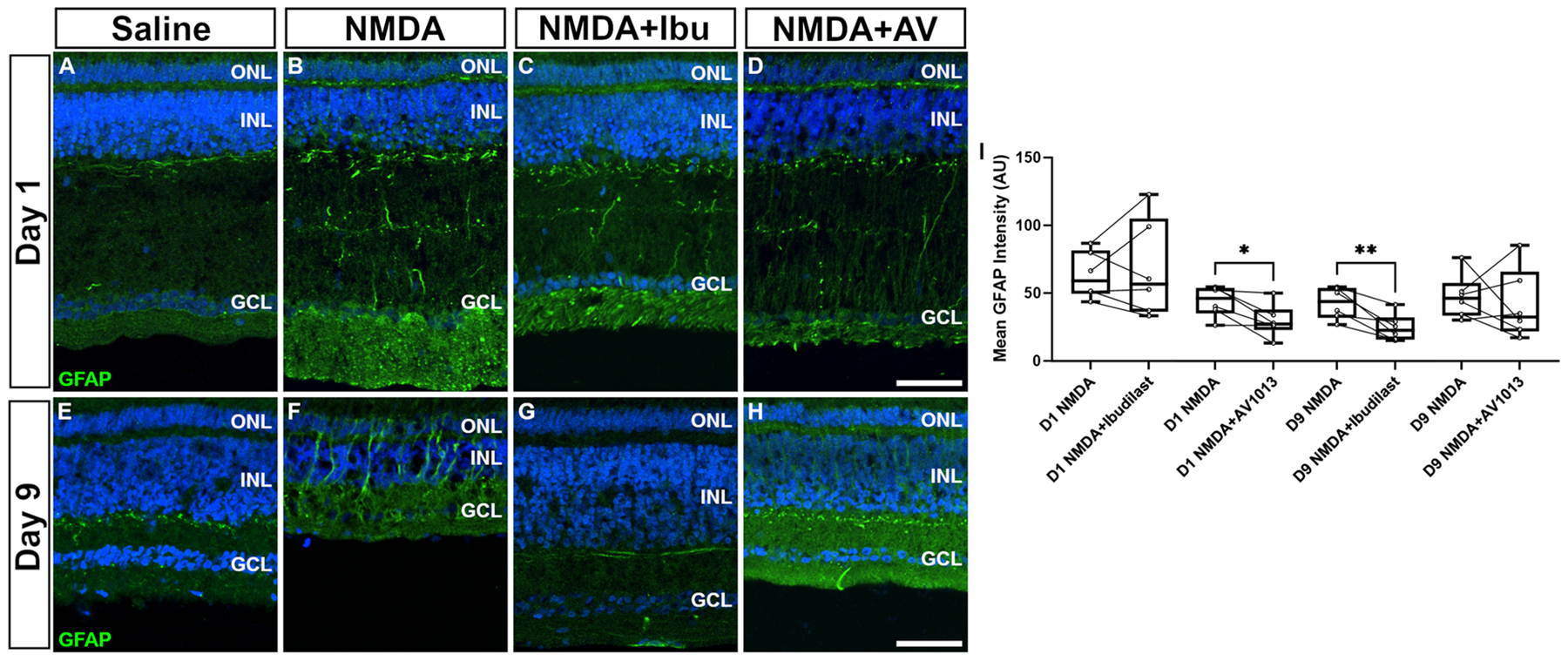
Ibudilast blocks retinal gliosis in retinal damage. Representative images of GFAP stained retinal sections from eyes injected with NMDA (A,D), NMDA-+ibudilast (B,E), or NMDA+AV1013 (C,F) at D1 (top panels) or D9 (lower panels). Mean GFAP intensity quantification (±SD) is shown in G. Each dot represents one biological replicate (n = 6/group). Significance of difference (*p < 0.05, **p < 0.01) was determined using a paired *t*-test. The calibration bar (50 μm) in panel C applies for all other images. Abbreviations: ONL – outer nuclear layer, INL – inner nuclear layer, GCL – ganglion cell layer.

Our *in vitro* results prompted us to test the effect of MIF inhibition on *in vivo* preclinical retinal animal models. We first sought to test the effect of ibudilast on retinal gliosis *in vivo*. The intermediate filament protein GFAP increases after retinal detachment and is a marker for gliotic changes important to the formation of excessive retinal scarring in PVR formation [37,38]. The chick eye is well established model for human eye disease including RD and excitotoxic damage due to its low cost, large size, cone-rich retina, and the presence of a macula equivalent (area centralis) [39,40]. We utilized our established chick excitotoxic eye injury model to induce gliosis in the eye using a N-methyl--D-aspartate (NMDA) intravitreal injection and evaluated the impact of intravitreal ibudilast and AV1013 delivery on retinal GFAP levels. As expected, excitotoxic damage increased GFAP intensity significantly (Fig. 6B). At nine days following NMDA injury, GFAP intermediate filament staining can be seen throughout the retinal layers (Fig. 6F). Interestingly ibudilast treatment significantly decreased retinal gliosis at day 9, when gliosis was at its peak, as measured by GFAP immunofluorescence (Fig. 6G). AV1013 showed an effect at inhibiting gliosis at day 1, however, that is when GFAP expression was minimal compared to day 9 (Fig. 6D & H).

### 3.7. Safety profile

Ibudilast was selected for further *in vivo* study since it is used clinically in Japan and is being evaluated in clinical trials in the US [22,41], while to our knowledge, AV1013 is not currently being developed for clinical use.

Our previous studies have shown that intravitreal ibudilast is safe to use *in vivo* [17]. Using a combination of cellular (TUNEL), structural (SD-OCT retinal layer thickness), and functional (ERG) studies we have shown that ibudilast does not induce any toxicity in the chick retina upon intravitreal injection of the maximum soluble concentration [17].

**Fig. 7.**
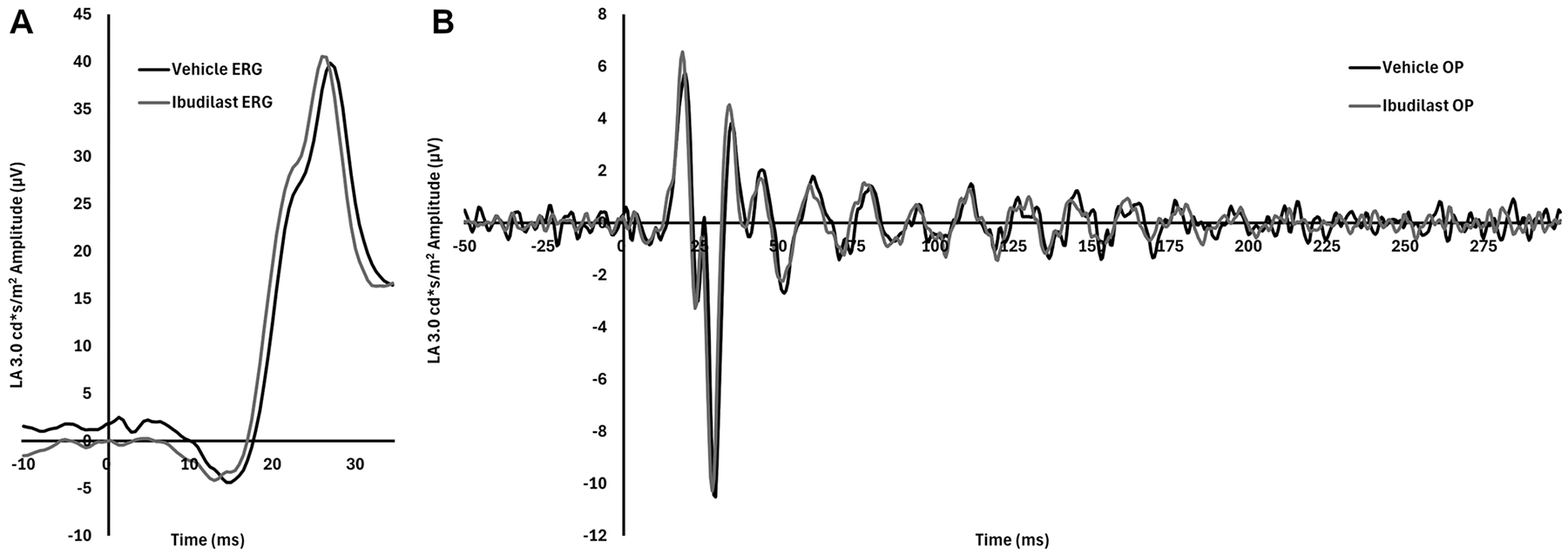
ERG amplitudes unchanged with ibudilast treatment. Rabbit ERG was performed 24 hrs after intravitreal injection of 150ug/50ul ibudilast (black line) in one eye and 50 μl saline vehicle (grey line) in the fellow eye. LA ERG (A) and oscillatory potential (B) at 3.0 cd*s/m^2^ plots are shown as average of 2 subjects.

We aimed to extend our toxicity studies to rabbits since the rabbit is the most commonly used preclinical model for testing ophthalmic drugs, and it is our animal model for establishing *in vivo* PVR. Light-adapted (LA) ERG was used to evaluate rabbit retinal function after 150 μg/50 μl ibudilast injection. ERGs were performed both pre-treatment and post-treatment. A-wave, b-wave, and oscillatory potentials were all analyzed. Ibudilast was injected intravitreously in the left eye and vehicle was injected in the right eye. There was no reduction in retinal function with ibudilast intravitreal injection compared to vehicle controls (Fig. 7). The LA ERG (Fig. 7A) and oscillatory potential plots (Fig. 7B) for both ibudilast and vehicle control are almost superimposed which indicates no negative effect on rabbit retinal function by ibudilast. Systemic toxicity was also evaluated by necropsy in rabbits after intraocular injection. Three 24 hr post-intravitreal injection rabbit necropsies were performed by the veterinary core facility. One rabbit was injected with a saline vehicle to serve as a control and two rabbits were injected with ibudilast. No major differences, such as ocular inflammation, were noted between the control and treatment groups (data not shown).

In summary, these data indicate that ibudilast does not induce any toxic effect when injected intraocularly at the maximum drug dose (0.15 mg/50 μl) used.

### 3.8. Pharmacokinetics in chick and rabbit

#### 3.8.1. Chick

**Fig. 8.**
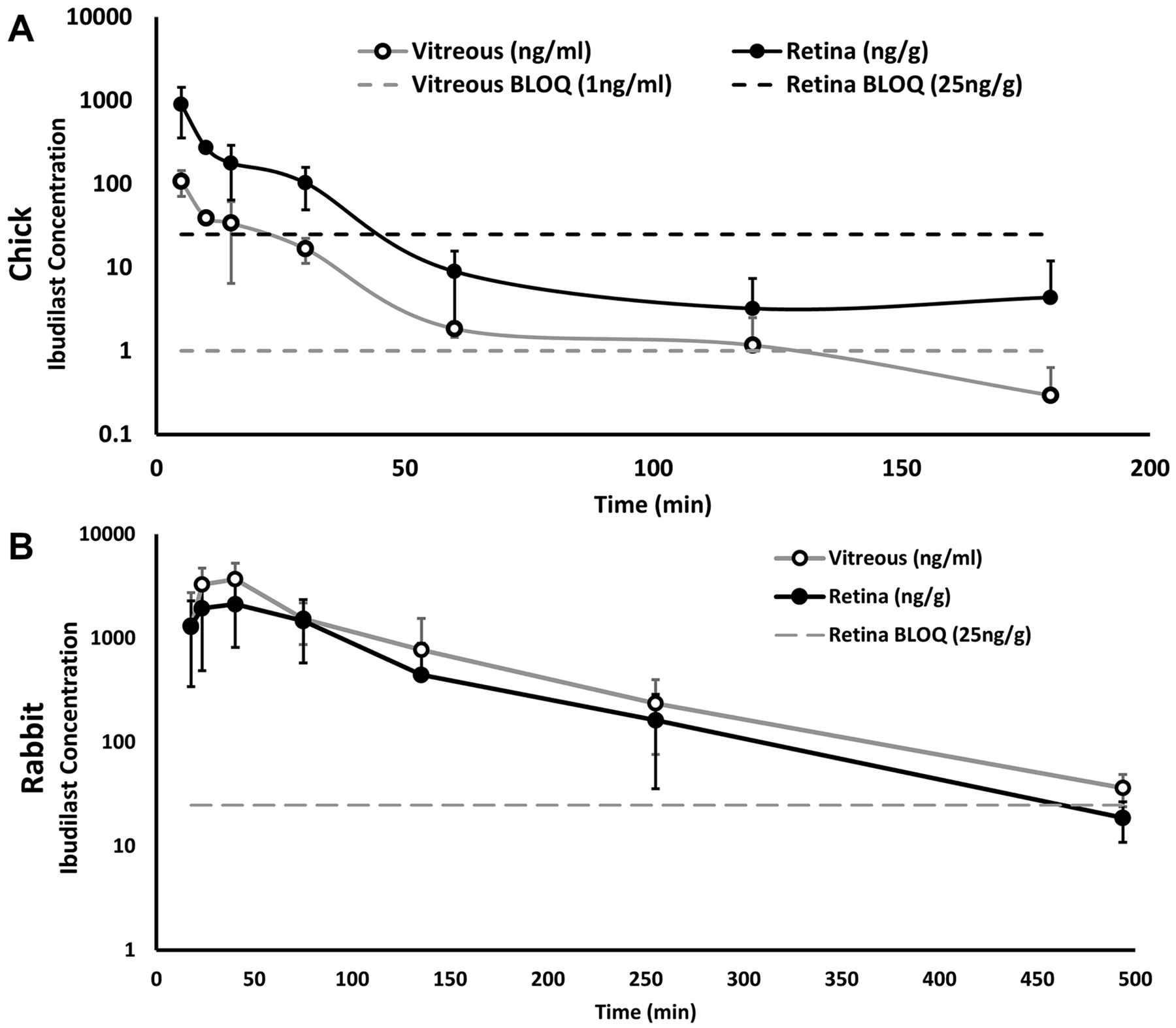
Pharmacokinetic (PK) studies of ibudilast in the chick and rabbit model. PK results of ibudilast injected into the vitreous of chicks (A) and rabbits (B) for ibudilast concentrations in the vitreous and the retina. Y-axis is log scaled and error bars that would fall below 0 are excluded. Vitreous ibudilast concentrations are represented by the grey line with the BLOQ (Below limit of quantification) represented as a grey dashed line. Retina ibudilast concentrations are represented by a black line with the BLOQ represented by a black dashed line (only shown in A). Data points under the BLOQ line are shown for thoroughness but should be disregarded for analysis.

To study the pharmacokinetics of ibudilast we started with a preliminary PK study in chick following a single intravitreal injection. PK analysis was performed to evaluate drug levels in the vitreous and retina at 3 min, 10 min, 30 min, 1 h, 2 h, 4 h, 8 h, 24 h, and 48 h post-injection. The measured ibudilast was identified at early timepoints in the retina and vitreous. The vitreal ibudilast half-life was measured to be 26.28 min and the retinal half-life was 31.07 min (Fig. 8A). Ibudilast concentration fell below the lower limit of quantification (LLOQ) after the 2 h time point. These results indicate rapid clearance of ibudilast from the eye.

Building on the pilot study data, we performed an extended PK study in ibudilast treated chicks (n = 4/group) focusing on the earlier time-points: 5 min, 10 min, 15 min, 30 min, 60 min, 120 min, 180 min, 480 min, and 1440 min. Retina and vitreous were collected from each subject. We also collected retina and vitreous from the fellow eyes at each timepoint to check for drug presence in the contra eye. Blood and plasma were also collected and analyzed. The injected eye vitreous and retina were averaged and plotted as log ng/mL against time (min) (Fig. 8A). The half-life was determined to be comparable to the previous pilot study. All tests performed on the fellow eyes and plasma were below the LLOQ (not shown).

#### 3.8.2. Rabbits

Since ibudilast had a short half-life in chick vitreous and retina following intravitreal injection, we focused on early timepoints for rabbit PK sample collection. A total of 12 rabbits were used to perform the PK study. Rabbits received an intraocular injection of ibudilast and were euthanized at various timepoints to measure drug concentrations in the vitreous, retina, and plasma (Fig. 8B). Tissue was collected 5, 10, 30, 60, 120, 240, and 480 min post-injection. The half-life of a single intravitreal injection of 150 μg/50 μl (3 mg/mL concentration) of ibudilast in the rabbit was determined to be 22 min in the vitreous and 78 min in the retina (Fig. 8B). No ibudilast was detected in the plasma after ocular injection.

### 3.9. MIF inhibition in rabbit PVR model

**Fig. 9.**
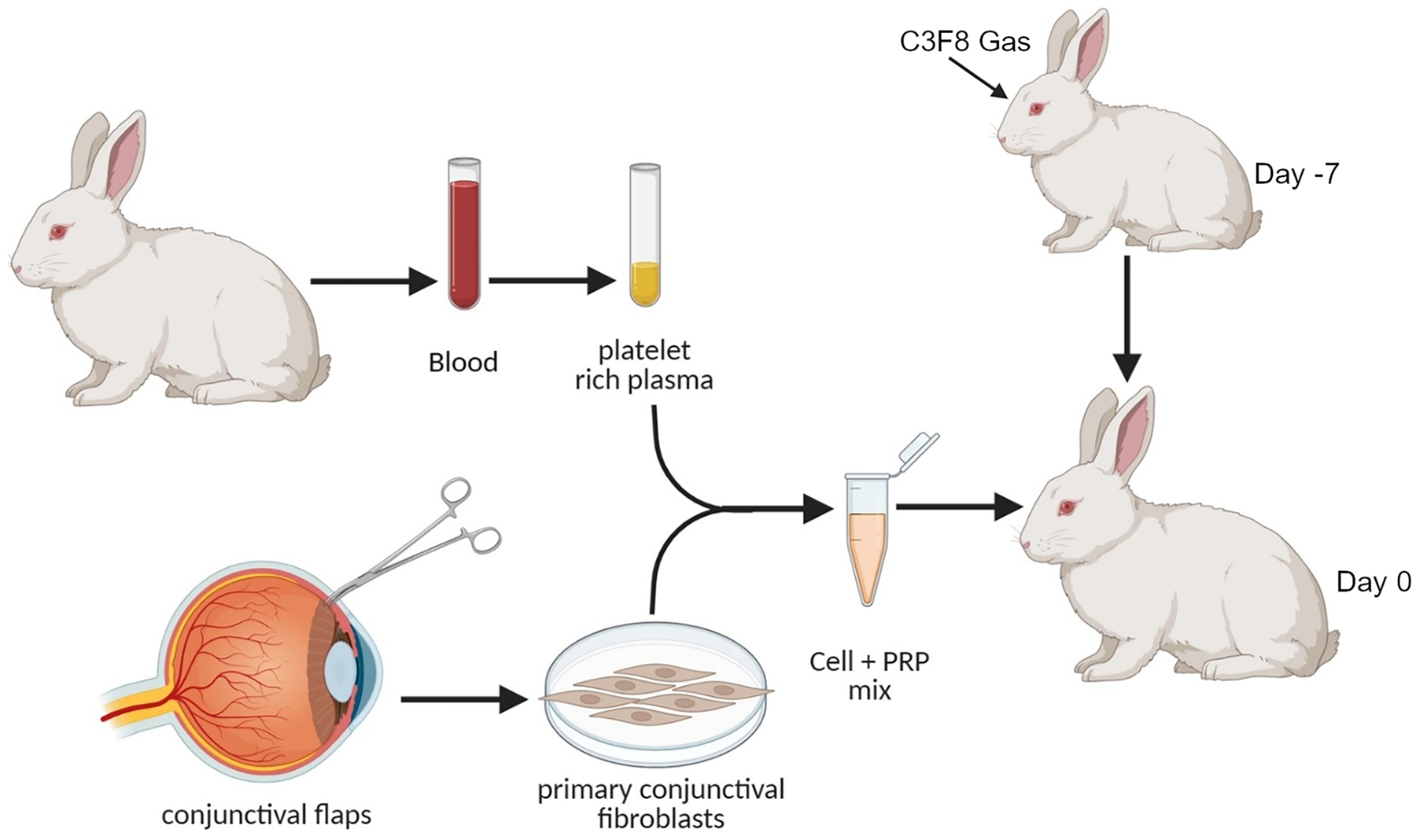
The Rabbit PVR model.

**Fig. 10.**
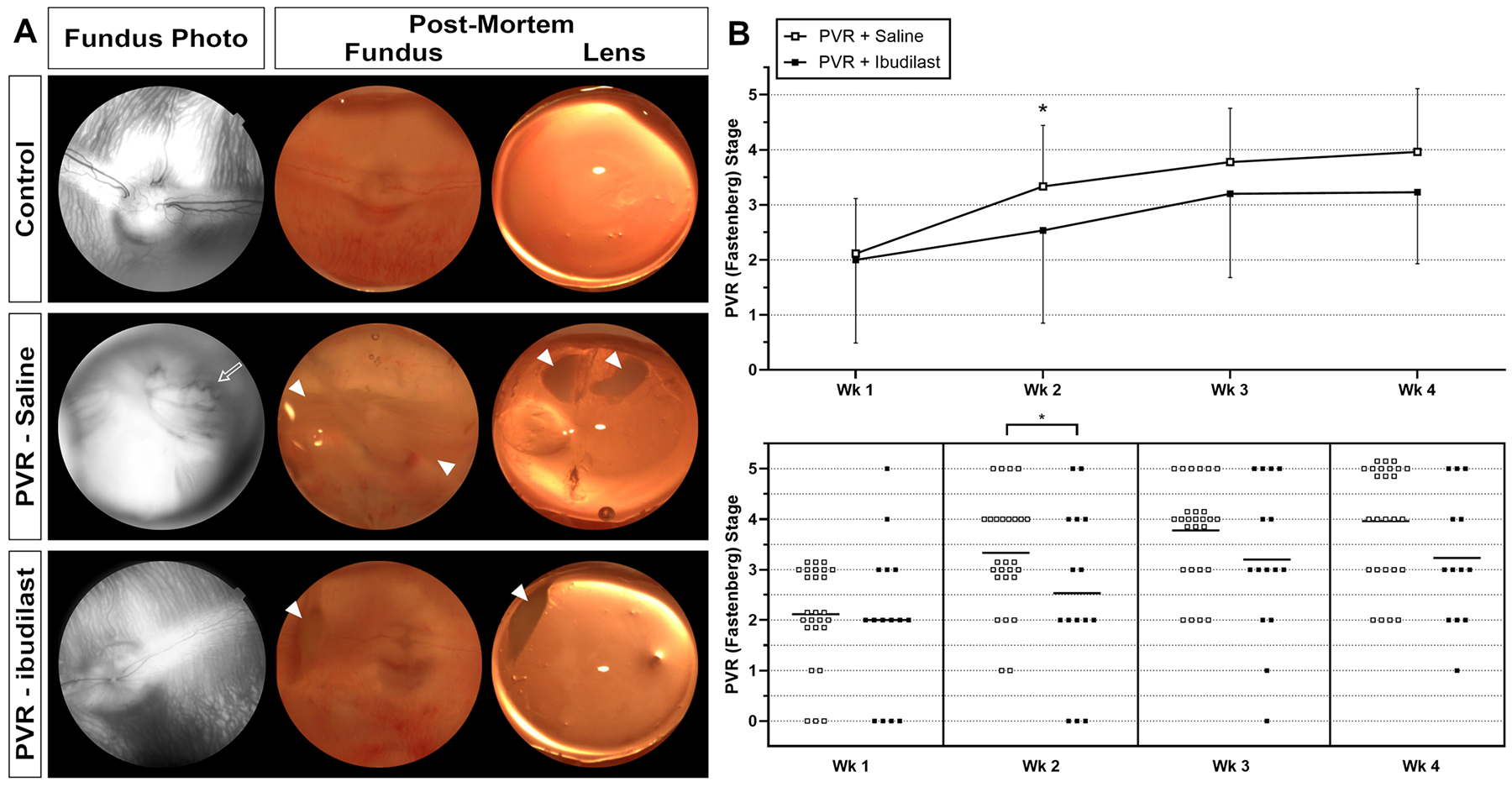
PVR progression in the in vivo rabbit model and the effect of ibudilast. A. Representative images of normal eyes (upper row) showing healthy appearance as imaged by fundus camera in live animal and post-mortem images of retinal fundus and lens. PVR progression is demonstrated in the PVR-saline eyes (second row) and is characterized by regions of retinal detachment with vascular tortuosity (open arrow). Post-mortem images show regions of fibrosis (closed arrow heads) and retinal detachment in the fundus. Fibrosis is also seen on the posterior lens capsule. Ibudilast treatment in PVR (lower row) shows preserved medullary rays, less fibrosis (closed arrow heads), and no RD. B. Progression of PVR in rabbits is assessed using Fastenberg staging and graphed over a period of 4 weeks. Ibudilast effect (closed squares) vs saline vehicle (open squares) is shown. Asterisks represent p < 0.05.

Our findings that showed the effectiveness of MIF inhibition at attenuating the pathogenesis of PVR *in vitro*, inhibiting gliosis *in vivo*, and favorable safety profile with intravitreal injection motivated us to investigate MIF inhibition effect on experimental PVR *in vivo*. The rabbit PVR model is well established and characterized in eye research, and it is a major preclinical model for ocular drug testing. Fig. 9 and the experimental section details the process of establishing the rabbit PVR model by injecting a C3F8 gas bubble followed by a mix of rabbit primary conjunctival fibroblasts with platelet rich plasma. Through masked grading of the clinical exam using the Fastenberg staging, the OCT b-scan, *en face* fundus imaging, and ERG we were able to confirm the development of PVR over the time period tested (5 weeks). Images of control and PVR affected eyes are shown in Fig. 10A.

We then aimed to test the ability of ibudilast to modulate the severity of PVR. Two doses of 50 μl of 3 mg/mL ibudilast were injected intravitreally: the first was administered immediately after the cell and platelet-rich plasma injection and the second followed three days later. The fellow eye was left untouched and used as a control. Half of the PVR rabbits (17 rabbits) were injected with intravitreal ibudilast, and the other half (17 rabbits) were injected with saline as a control. Rabbits were evaluated over a 5 week period at day 1, 3, 7, 14, 21, and 28. PVR was graded in a masked fashion, according to the Fastenberg scale of classification [42].

Interestingly and consistent with our *in vitro* findings, ibudilast treatment conferred protection from the severity of the PVR disease the rabbit model, compared to saline control injection (Fig. 10). A statistically significant decrease in PVR severity (grading) was observed on week 2 in the ibudilast group compared to the saline group (*p-value* = 0.039). Additionally, a higher grade PVR (4 & 5) was observed more often in the saline group compared to the ibudilast group throughout the course of study. At the end of our studies (week 4) two thirds of saline-treated eyes were in grade 4 or 5 compared to less than half of the ibudilast treated eyes. These results are summarized in Fig. 10. Overall, our finding suggests that ibudilast confers protection from PVR progression in the rabbit model.

## 4. Discussion

The lack of approved pharmacologic therapies for the prevention or treatment of proliferative vitreoretinopathy underscores the significance of identifying targets that can regulate the multiple pathogenic processes of this disease [43]. In this study we have demonstrated the proof of concept that MIF inhibitors have potential to combat PVR. MIF inhibition was able to block the multiple steps critical to PVR pathogenesis *in vitro*. Both ibudilast and its analogue AV1013 were equally effective at blocking cell proliferation, EMT, migration, and gel contraction. *In vivo*, intravitreal ibudilast was well tolerated, counteracted gliosis in chick eyes, and prevented PVR progression and severity in a rabbit *in vivo* PVR model.

MIF has been implicated in the pathogenesis of several fibrotic diseases. MIF levels increase significantly in the thioacetamide-induced liver fibrosis model [44] and the bleomycin induced pulmonary fibrosis model [28]. Overexpression of MIF in genetically modified mice resulted in collagen IV accumulation, a marker for fibrosis, in the kidneys [15]. MIF inhibition has been found to counteract other conditions with a fibrotic component. For example, MIF inhibition leads to a decrease in cicatricial disease progression, such as bleomycin pulmonary fibrosis using genetic MIF knock-down [28] or pharmacological inhibitors like ISO-1 [45]. In a rat model for joint contracture, MIF promoted fibroblast activation [46], while injection of MIF inhibitor 4-IPP significantly suppressed joint capsule inflammation and fibrosis. Together, these studies suggest that MIF inhibition is a new therapeutic target in fibrotic conditions. Herein we show that MIF inhibition should be further investigated in ocular fibrotic disease.

Although our data demonstrate that MIF inhibition effectively attenuates multiple steps critical to PVR pathogenesis, the precise mechanisms by which MIF drives ocular fibrosis remain to be fully elucidated. In the progression of fibrotic lung diseases, MIF is thought to act through both immune modulation and direct effects on structural cells like fibroblasts [16]. Mechanistically, MIF signals primarily through the CD74 receptor, often in complex with CD44 and chemokine receptors, activating downstream pathways like MAPK/ERK, PI3K/Akt, and NF-κB. These signaling cascades regulate the survival and activation of immune cells (especially macrophages), fibroblasts, and epithelial cells [47].

MIF has been implicated in the pathogenesis of many ocular diseases [48]. In retinal pigment epithelial cells *in vitro* MIF has been shown to promote cell proliferation, migration, and contraction- key features of fibrotic membrane formation [49]. These studies also demonstrated that MIF increases the expression of proinflammatory cytokines like IL-6 and MCP-2. MIF also plays a role in neovascularization and inflammation in proliferative diabetic retinopathy (PDR). Analysis of epiretinal fibrovascular membranes from PDR patients showed elevated levels of MIF and its receptor CD74 in those membranes [50]. Moreover, the study found significant positive correlations between the levels of MIF and the angiogenic activity in PDR as measured by neovascularization markers, which suggests a potential role for MIF in PDR pathogenesis. Consistent with this, MIF-knockout mice showed reduced retinal neovascularization compared to wild type during hypoxia-induced mouse model of retinal neovascularization [51]. This was associated with reduced expression of VEGF and inflammatory mediators, as well as decreased recruitment of endothelial progenitor cells and microglia. In the context of PVR, it remains unclear whether MIF primarily exerts its effects through modulation of the inflammatory microenvironment, direct actions on RPE and glial cells, or a combination of both. Recent studies utilized transcriptomic analysis and genetic tracing to reveal interesting roadmap to fibrosis formation during proliferative vitreoretinopathy [52,53]. Dissecting the role of MIF in cell-type–specific contributions and downstream signaling pathways will be important for refining therapeutic strategies and optimizing target specificity.

Although we found that multiple MIF inhibitors blocked PVR mechanisms in our *in vitro* studies, we decided to focus on ibudilast in our *in vivo* PVR model because of the drug’s clinical relevance and translational potential. Ibudilast is already used clinically in Japan and South Korea for the treatment of asthma, post-stroke complications, and allergic conjunctivitis [54,55]. In the US, ibudilast is being evaluated in clinical trials for neurodegenerative diseases like multiple sclerosis and amyotrophic lateral sclerosis [19,22,41,56].

In a separate ocular disease model involving excitotoxic injury, we found that ibudilast is protective to retinal neurons [17]. In that model the PDE inhibitory properties of ibudilast seem to be critical to its neuroprotective effect. This conclusion was supported by the observation that the analogue AV1013, which lacks PDE inhibition, did not preserve retinal neurons. Interestingly in the current study AV1013 demonstrated protective effects in the *in vitro* PVR model. PDEs modulate diverse cellular processes by regulating cyclic nucleotide signaling pathways (cAMP and cGMP), which play key roles in inflammation, cell survival, apoptosis, and synaptic function. Growing evidence suggests that PDEs are implicated in the pathophysiology of many neuronal and inflammatory diseases [56]. Targeting PDEs, especially PDE4, has proven to be an effective therapeutic strategy for multiple diseases with inflammatory and neuroinflammatory components [57]. This raises the question of whether PDE inhibition contributes to ibudilast’s protective mechanism in PVR as well. We are currently evaluating the effect of PDE inhibitors on retinal excitotoxic damage. Evaluating these compounds in the PVR model may provide valuable insights into the mechanism underlying ibudilast’s protective action in PVR. Although AV1013 was not successful in preserving retinal neurons in excitotoxic damage, both it and ibudilast were effective at blocking the multiple steps of PVR. This provides evidence that MIF is a good candidate to investigate in PVR and additional MIF inhibitors should be evaluated.

Methotrexate is a promising agent for PVR and is being used off label in clinical settings. It is under investigation in trials to clarify its role in both treatment and prevention of PVR [58]. Methotrexate efficacy is thought to stem from its ability to suppress both proliferative and inflammatory components of the disease [59]. Given that MIF is a key modulator of inflammation and cell survival, it is plausible that MIF inhibition could complement methotrexate’s mechanism of action. Ibudilast, known to inhibit MIF in addition to PDEs, may therefore offer a dual mechanism of protection in PVR—targeting both inflammatory and neurodegenerative pathways. Investigating the interplay between MIF inhibition and methotrexate treatment could uncover synergistic effects and inform combination therapies for improved PVR management.

Several limitations of the present study should be acknowledged. First, while the chick and rabbit models capture key features of PVR pathogenesis, including gliosis, membrane formation, and tractional changes, they do not fully capture the complexity and heterogeneity of human PVR. Second, although comprehensive safety assessments demonstrated that intravitreal ibudilast was well tolerated in vitro, in the chick eye, and in the rabbit model, these findings reflect short term exposure and may not fully capture the safety profile associated with repeated or long-term dosing. While these results are consistent with prior reports describing favorable in vivo tolerability of ibudilast, extended safety studies will be required to support chronic ocular use in clinical settings. Third, pharmacokinetic analyses revealed that ibudilast exhibits a relatively short half-life in the retina following intravitreal administration. This rapid clearance may limit sustained drug levels and therapeutic efficacy when delivered as a single injection, particularly in a disease such as PVR that evolves over time. Consequently, the current formulation may not represent the optimal mode of delivery for clinical translation. Future efforts focused on formulation optimization will be critical to extend intraocular drug exposure, enhance pharmacodynamic effects, and improve clinical feasibility. Fourth, while ibudilast was selected for in vivo studies based on its established clinical use and translational potential, it is a pleiotropic compound that inhibits both MIF and PDE. As a result, the relative contribution of MIF inhibition versus PDE inhibition to the observed protective effects in PVR cannot be fully delineated in the current study and needs further investigation. Finally, this study primarily evaluated early intervention; the efficacy of MIF inhibition in established or advanced PVR remains to be determined.

In summary, our study identifies MIF as a promising therapeutic target in PVR and demonstrates that pharmacologic inhibition with ibudilast attenuates key pathogenic mechanisms in preclinical models. By targeting both inflammatory and fibrotic pathways, MIF inhibition represents a rational and translational approach for PVR management. These findings warrant further mechanistic investigation and support continued development of MIF-targeted therapies for ocular fibrotic disease.

## Data Availability

No data was used for the research described in the article.
